# Larva migrans syndrome caused by *Toxocara* and *Ascaris* roundworm infections in Japanese patients

**DOI:** 10.1007/s10096-016-2693-x

**Published:** 2016-06-06

**Authors:** A. Yoshida, A. Hombu, Z. Wang, H. Maruyama

**Affiliations:** Division of Parasitology, Department of Infectious Diseases, Faculty of Medicine, University of Miyazaki, Kihara 5200, Kiyotake-cho, Miyazaki City, Miyazaki 889-1692 Japan

## Abstract

Larva migrans syndrome (LMS) caused by *Toxocara* and *Ascaris* roundworms is generally believed to be more common in children, while a report from Japan suggests that it is more common in adults. We conducted a large-scale retrospective study to confirm these findings and to clarify what caused the difference between Japan and other countries, to reveal overlooked aspects of this disease. The clinical information of 911 cases which we diagnosed as *Toxocara* or *Ascaris* LMS during 2001 and 2015 was analysed. Information used included age, sex, address (city or county), chief complaint, present history, dietary history, overseas travelling history, medical imaging findings and laboratory data (white blood cell count, peripheral blood eosinophil number and total IgE). The sex ratio of the disease was 2.37 (male/female = 641/270). The number of patients not younger than 20 years old were 97.8 and 95.1 % among males and females, respectively. Major disease types were visceral, ocular, neural and asymptomatic. The visceral type was more prevalent in older patients, while younger patients were more vulnerable to ocular symptoms. More than two-thirds of the patients whose dietary habits were recorded had a history of ingesting raw or undercooked animal meat. LMS caused by *Toxocara* or *Ascaris* is primarily a disease of adult males in Japan, who probably acquired infections by eating raw or undercooked animal meat/liver. Healthcare specialists should draw public attention to the risk of raw or undercooked animal meat in Europe as well.

## Introduction

Larva migrans syndrome (LMS) is a typical zoonosis caused by parasitic worms which cannot mature in humans. Although various kinds of parasites could cause LMS [1–5], the most popular and cosmopolitan species are *Toxocara canis* and *T. cati* [6, 7], which are native to dogs and cats, respectively. In addition to *Toxocara* spp., LMS caused by *Ascaris suum* has also been reported [8–10]. *Toxocara canis*, *T. cati* and *A. suum* are all large intestinal roundworms belonging to the family Ascarididae, so LMS caused by these parasites could be referred to as ascarid LMS.

Humans acquire infection with these ascarid nematodes by ingesting embryonated eggs contaminating the soil or vegetables, or eating raw/undercooked paratenic host meat, such as chicken and beef, in which infective larvae reside. After the ingestion of eggs or infected meat, third-stage larvae invade intestinal mucosa, and migrate to various tissues and organs through blood or lymph vessels, eliciting potent immune responses with eosinophilic inflammation. Typical and classical target organs are the liver and lungs, causing symptoms classically described as visceral larva migrans (VLM). Eyes (ocular larva migrans; OLM), brain and spinal cord (neural larva migrans; NLM) could also be affected [6, 7, 11].

A large body of literature on human toxocariasis, infection with *T. canis* or *T. cati*, is available from European and North American countries, in which toxocariasis is considered to be more common in children [12–16]. Because children often visit egg-contaminated areas and ingest dirt as a result of their play habits and hygiene, they have a greater chance of contracting infections. Geophagia and owning a dog or cat are believed to be other factors that increase the risk of infection [13, 17, 18]. However, a report from Japan dealing with a relatively small number of patients suggests that toxocariasis in Japan is more common in adults [19].

**Fig. 1:**
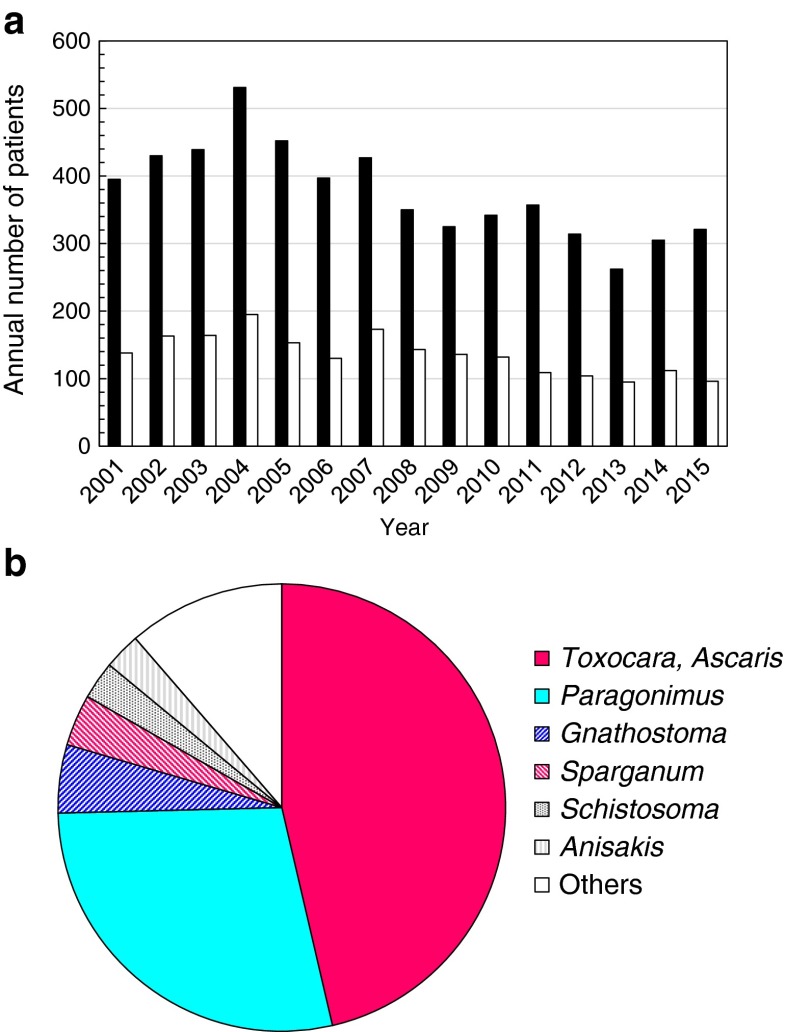
Diagnosis of parasitic infections at the Division of Parasitology, Department of Infectious Diseases, University of Miyazaki. **a** Annual number of examined patients (■) and positive patients (□). **b** Major parasitic diseases in 2001–2015

Since the early 1990s, our laboratory has been one of the antibody test centres for parasitic diseases in Japan. Each year, we test hundreds of sera and body fluids, such as pleural effusion, cerebrospinal fluid and vitreous humour, for anti-parasite antibodies in order to help physicians to make a diagnosis. As a result, we assist in diagnosing approximately 100 parasitic infections every year to find that LMS caused by *Toxocara* or *Ascaris* is the leading parasitic disease (Fig. 1). For the clarification of the epidemiology and the nature of this disease among Japanese people, we conducted a retrospective case study of this seemingly overlooked syndrome over the last 15 years, from 2001 to 2015. Based on 911 clinical cases, we found that ascarid LMS in Japanese people appears to have a unique characteristic as compared to that in European and North American people. Our data should constitute a scientific basis for the effective prevention of this important parasitic zoonosis.

## Materials and methods

### Patients analysed in the present study

During 2001 and 2015, samples of 5532 patients, which mainly consisted of sera, were tested and 955 patients were diagnosed as having *Toxocara* or *Ascaris* infections. During the study period, we experienced only a single patient in which a parasite larva was directly demonstrated. In this case, a biopsy specimen was taken from the spinal cord of a 33-year-old female patient who had suffered from paresthesia in the left shoulder. Pathology revealed a *T. canis* larva in histological sections. All other cases were diagnosed based on the results of antibody tests without direct worm demonstration.

However, laboratory personnel who were responsible for the antibody test and diagnosis changed more than a couple of times during this period, affecting assay procedures and decision protocol. Therefore, we re-examined the clinical history and serum antibodies of all 955 patients to select unambiguous cases for analysis. As a result, 911 patients were diagnosed as having *Toxocara* and/or *Ascaris* infection under the present criteria (see below).

We excluded patients who expelled or vomited adult *Ascaris* worms (17 cases in total) from the present study, because they were not considered to have LMS.

When a patient met at least one of the following criteria, we judged him/her as having LMS caused by *Toxocara* or *Ascaris* infection:

1. Positive binding of serum to *Toxocara* and/or *Ascaris* antigen with peripheral blood eosinophilia.
2. Tissue eosinophil infiltration revealed by pathology of biopsy specimen with positive binding of serum or local fluid, e.g. cerebrospinal fluid (CSF), pleural effusion, ascites, broncho-alveolar lavage fluid (BALF), vitreous humour fluid and pericardial fluid.
3. Medical imaging techniques, such as chest X-ray, computed tomography (CT), magnetic resonance imaging (MRI) and ultrasonography (US), strongly suggested worm infections, e.g. multiple nodular lesions in lungs/liver, swelling in spinal cord, with positive binding of serum or local fluid.

When serum antibodies were positive but no sign of ongoing infections could be seen, e.g. no eosinophilia, no abnormal imaging, no symptoms, no pathology, we considered this as past infection and excluded it from the present study.

### Antigen preparation for antibody test

*Ascaris suum* adult worm somatic antigen was prepared as follows. Adult worms, collected from infected pigs at a local abattoir, were homogenised in 0.15 M phosphate-buffered saline (PBS, pH 7.2), extracted by magnetic stirring at 4 °C overnight and centrifuged at 10,000 × g for 10 min at 4 °C. The supernatant was used as *Ascaris* soluble worm antigen preparation (As-SWAP).

Excretory/secretory (ES) antigen of *T. canis* larvae (Tc-ES) was prepared as previously described [20]. Briefly, *T. canis* third-stage larvae were hatched mechanically and cultured in RPMI-1640 (Wako, Osaka, Japan) containing 100 μg/ml of streptomycin, 100 U/ml of penicillin and 250 ng/ml of amphotericin B (Gibco, Rockville, MD, USA), at 37 °C. The supernatant was collected weekly and concentrated by ultrafiltration (Amicon Ultra-15 3K, Millipore, Billerica, MA, USA).

For ES antigen of *A. suum* larvae (As-ES), *A. suum* third-stage larvae were collected by Baermann’s method from the lungs of infected male Japanese white rabbits (Kyudo, Kumamoto, Japan), which were orally inoculated with 1.5 × 10^5^ embryonated eggs. Larvae were then cultured in RPMI-1640 and culture supernatant was collected and concentrated as described above.

### Enzyme-linked immunosorbent assay (ELISA)

Sera and body fluid samples were tested for specific antibodies in an enzyme-linked immunosorbent assay (ELISA). Antigen preparations used in the ELISA were As-SWAP, Tc-ES and As-ES. In our standard procedure, samples were first tested for the binding to As-SWAP and Tc-ES, and in non-typical cases, e.g. eosinophilic enteritis and eosinophilic myocarditis, samples were further tested in a Western blotting kit (LDBIO Diagnostics, Lyon, France) for confirming toxocariasis-specific band patterns. In 120 randomly selected samples, we compared binding to Tc-ES and As-ES to determine *Toxocara* LMS or *Ascaris* LMS.

In the ELISA, wells of microtitre plates (Nunc, Roskilde, Denmark) were coated overnight at 4 °C with 2 μg/ml of As-SWAP or 1 μg/ml of Tc-ES or As-ES in 0.05 M carbonate-bicarbonate buffer (pH 9.6). The wells were washed with PBST (PBS containing 0.05 % Tween 20), blocked with 1 % casein (Nakarai Tesque, Kyoto, Japan) and incubated with diluted sera (1:900 and 1:2700) for 1 h at 37 °C. After washing with PBST, binding of antibodies was detected with horseradish peroxidase conjugated rabbit anti-human IgG (Dako, Glostrup, Denmark). For colour development, ABTS (KPL, Gaithersburg, MD, USA) was added and incubated at room temperature for 30 min in the dark. The optical density at 405 nm was read in a Microplate Reader (Bio-Rad Laboratories, Hercules, CA, USA). Based on negative control serum data, we set a cut-off point at 0.2 for 1:900 diluted sera, pleural effusion and ascites. For body fluids such as CSF and vitreous humour fluid, optical density more than 0.1 at 1:30 dilution was judged positive.

### Disease type

The clinical information of individual patients was provided by the attending physicians on a form of consultation sheet, which contained age, sex, address (city or county), ethnic origin, chief complaint, brief summary of the present history, dietary history, overseas travelling history, medical imaging findings and laboratory data, including white blood cell (WBC) count (/μl), peripheral blood eosinophil number (/μl) and total IgE (IU/ml). Based on the available data, we divided patients into several disease types according to the affected organs.

In the present study, we did not categorise the ‘covert’ type, which is asymptomatic but shows abnormal imaging and/or laboratory data as reported in the literature [11, 21–24], because we believe that any sign of organ disorder, indicated by medical imaging or blood chemistry, should be considered ‘visceral,’ even if patients were asymptomatic:

1. Visceral larva migrans (VLM): Pulmonary symptoms such as cough, sputum, dyspnoea, chest pain and/or hepatic lesions revealed in medical imaging techniques, such as chest X-ray, X-ray CT, MRI or US.
2. Ocular larva migrans (OLM): Visual disturbance with a sign of uveitis and/or retinal nodular lesions with positive binding of serum or local fluid to As-SWAP or Tc-ES.
3. Neural larva migrans (NLM): Neurological disorders such as paresthesia, muscle weakness, urination disorder with or without medical imaging (CT, MRI) findings of the brain and/or spinal cord. Binding of serum or CSF to As-SWAP or Tc-ES.
4. Asymptomatic: No symptoms complained by patients nor any objective findings other than peripheral blood eosinophilia with high antibody titre against As-SWAP, Tc-ES or As-ES. In most cases, patients of this type were noticed upon regular medical check-up or follow-up examination for other chronic diseases, e.g. hypertension, diabetes mellitus, chronic kidney disease, asthma.
5. Miscellaneous: Symptomatic but not included in VLM, OLM or NLM. This type was further divided into several types. (1) Cardiac type was characterised with signs of cardiovascular involvement (congestive heart failure, myocarditis and pericarditis) with peripheral blood eosinophilia. In some cases, pathology demonstrated the eosinophil infiltration into the myocardium. (2) Gastrointestinal type showed diarrhoea and abdominal pain. Eosinophil infiltration in intestinal mucosa might be seen in biopsy specimens. (3) Cases with skin eruption (erythema and urticarial swelling) were referred to as cutaneous type. (4) Oedematous had oedema in extremities or other part of the body, showing no skin eruption.

### Statistical analysis

The correlation between disease type, sex ratio and age was analysed by the Pearson correlation coefficient. Residual analysis was used to compare peripheral blood eosinophilia or IgE level among the different disease types. Welch’s *t* test was employed to evaluate differences in the number of eosinophils and the concentration of IgE between groups. Differences in the infection positive rate in different geographical areas were evaluated with Chi-square tests. *p*-Values of less than 0.05 were considered statistically significant.

## Results

### Age and sex distribution

**Fig. 2:**
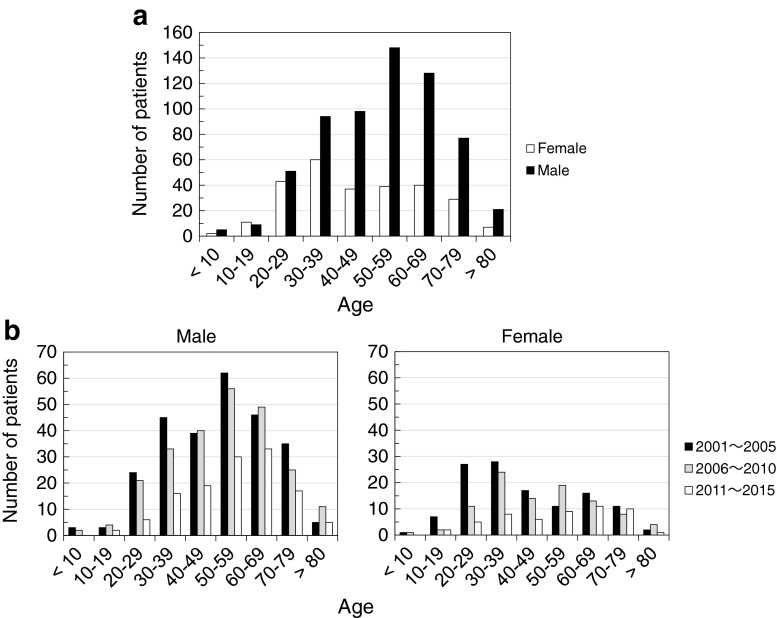
Age distribution of patients. **a** Overall age and sex distribution of 911 patients. **b** Number of male and female patients in 2001–2005, 2006–2010 and 2011–2015

Among 911 cases, there were 641 male patients and 270 female patients (sex ratio: 2.37). The number of patients not younger than 20 years old were 97.8 and 95.1 % among males and females, respectively. The number of children younger than 10 years old, on the other hand, was 7 in total (0.77 %) during the 15-year study period. The peak age group was 50s among males and 30s among females in the overall group of patients (Fig. 2a), though younger patients drastically decreased in the last 5 years (Fig. 2b). Thus, it was clear that ascarid LMS, caused by *Toxocara* or *Ascaris*, is primarily a disease of middle-aged males in Japanese patients, which is a major difference from the epidemiology in Western countries.

### Proportion of toxocariasis and ascariasis

**Fig. 3:**
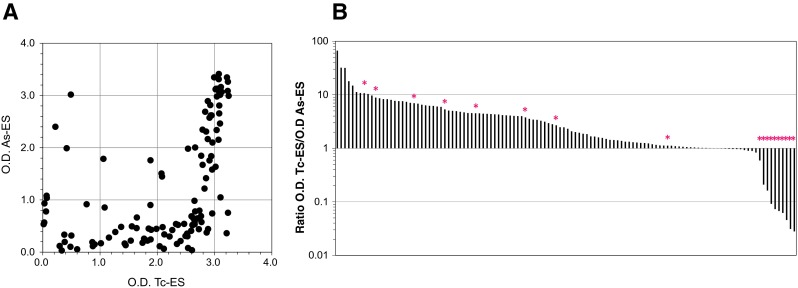
Binding of patient sera to larva ES antigens. One hundred and twenty randomly selected serum samples of ascarid LMS patients were examined for the binding to Tc-ES and As-ES in the ELISA. **a** Binding to Tc-ES (x-axis) and As-ES (y-axis). **b** Ratio of optical densities in the ELISA (Tc-ES/As-ES). *Negative in *Toxocara* Western blotting

In order to estimate the proportion of toxocariasis cases in the present study, we examined 120 randomly selected serum samples for the binding to Tc-ES and As-ES in a microtitre plate ELISA. As shown in Fig. 3a, most of the serum samples bound to Tc-ES more strongly than As-ES, with only a portion showing stronger binding to As-ES. These results suggested that most patients were infected with *Toxocara* nematodes. We then tested the samples in a commercially available *Toxocara* Western blotting kit to confirm the results of the ELISA. We found that the top ten samples which showed stronger binding to As-ES in the ELISA were all negative in *Toxocara*-specific Western blotting, strongly suggesting that these 8.3 % (10 in 120) were infected with *Ascaris* (Fig. 3b). Among the samples that showed stronger binding to Tc-ES in the ELISA, eight samples (6.7 %) were negative in Western blotting. Considering that the sensitivity of the kit may not be 100 % in our hands, we presumed that the proportion of toxocariasis in ascarid LMS cases in Japan was somewhere between 85.0 % (102/120) and 91.7 % (110/120).

### Disease type

In the present analysis, we recognised well-established major disease types: VLM, OLM, NLM and asymptomatic type, which, in our definition, showed no objective abnormalities other than peripheral blood eosinophilia and high antibody titre against As-SWAP, Tc-ES or As-ES. VLM was the most common, followed by OLM, asymptomatic and NLM (Table 1), which was in accordance with previous reports [6, 7]. In NLM, six cases were eosinophilic meningitis and most of the rest were myelitis at the cervical or thoracic level. MRI showed high signal intensity lesion on T2-weighted images (T2WI). Some of our cases have been reported previously [25, 26].

**Table 1:** Major and minor disease types of ascarid LMS

Disease type	Number of cases	Eosinophils (/μl)	IgE (IU/ml)
Visceral	352 (38.6 %)	3312 ± 353	3088 ± 451
Ocular	173 (19.0 %)	348 ± 86	688 ± 194
Neural	102 (11.2 %)	712 ± 139	1302 ± 259
Miscellaneous	135 (14.8 %)	4309 ± 613	2118 ± 389
Cardiac	33 (3.6 %)		
Gastrointestinal	25 (2.7 %)		
Cutaneous	41 (4.5 %)		
Edema	11 (1.2 %)		
Others	25 (2.7 %)		
Asymptomatic	149 (16.4 %)	3484 ± 499	4135 ± 636

**Fig. 4:**
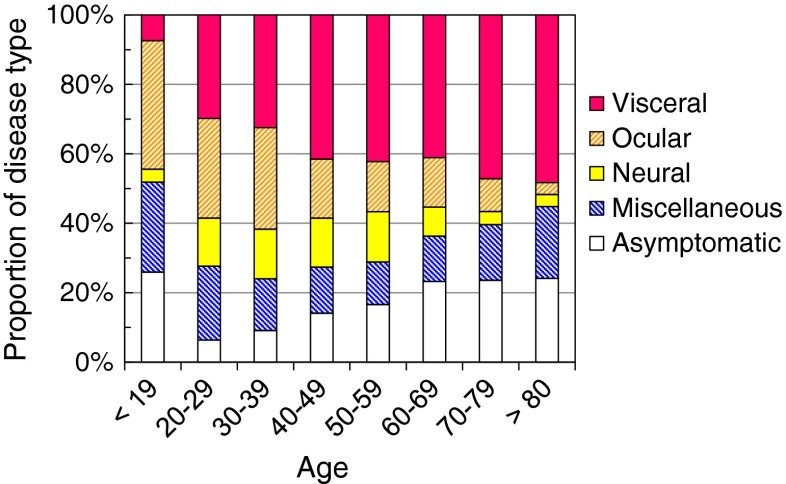
Proportion of disease types in different age groups

When the proportion of disease types was compared between different age groups, it turned out that OLM was more frequent in younger patients, while VLM was more prevalent in older patients (Fig. 4). The correlation coefficient for disease type and age was 0.97 for OLM and 0.89 for VLM. In other types, we did not find any significant correlation among different age groups. There was no statistical difference in the disease type distribution between male and female patients.

Of the rest of the above-described major types, 135 patients (14.8 %) presented with miscellaneous symptoms, which could be divided into cutaneous, cardiac, gastrointestinal and edematous types (Table 1). The cutaneous type in ascarid LMS is different from typical cutaneous larva migrans (CLM), which is caused by a migrating parasite larvae casing creeping eruptions. Skin eruptions seen in ascarid LMS were erythema and urticarial swelling.

Peripheral blood eosinophilia was prominent in VLM, asymptomatic and miscellaneous types, with the average exceeding 3000/μl (Table 1). Among these three groups, there was no statistical difference in the number of eosinophils. On the other hand, the average number of eosinophils in OLM and NLM was less than 1000/μl, significantly lower than the VLM, asymptomatic and miscellaneous groups. Between OLM and NLM, the eosinophil number of NLM was larger than that of OLM. A residual analysis indicated that the proportion of eosinophilia patients (>500/μl) increased significantly in the VLM, NLM, asymptomatic and miscellaneous types, though no significant increase was seen in the OLM group.

**Fig. 5:**
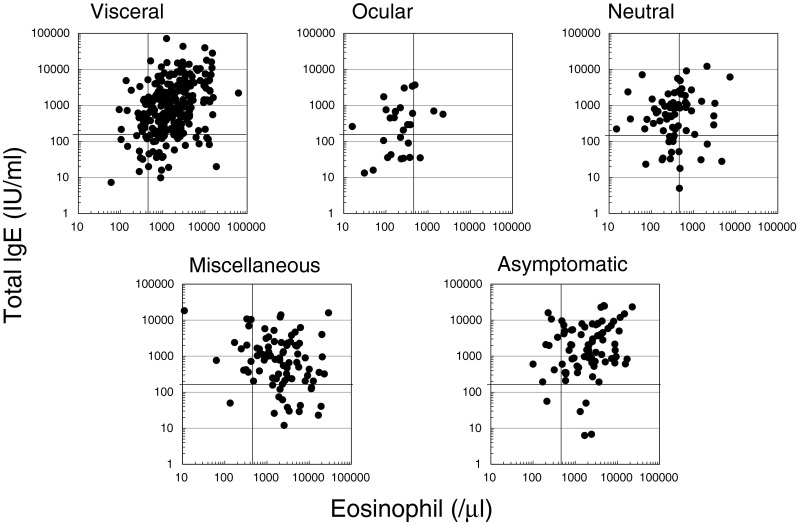
Relationship between serum IgE and peripheral blood eosinophilia in different disease types. The bars in the scatter graphs indicate normal values (eosinophils < 500/μl, IgE < 170 IU/ml)

As for the total IgE, the highest average concentration was seen in the asymptomatic group, followed by VLM, miscellaneous, NLM and OLM (Table 1). The IgE concentration in OLM was significantly lower than those of asymptomatic, VLM and miscellaneous, but no different to NLM. A residual analysis indicated that the proportion of high IgE patients (>170 IU/ml) increased significantly in the VLM, asymptomatic and miscellaneous types, though no significant increase was seen in the OLM and NLM groups. The relationship between eosinophilia and total IgE in each type is shown in Fig. 5.

### Dietary history

Because of the age distribution of ascarid LMS as described above, it was quite unlikely that infections with *Toxocara* and/or *Ascaris* in Japanese patients were associated with the ingestion of dirt as a result of play habits or geophagia, as in previous reports [17, 18]. Instead, the consumption of raw or undercooked animal meat seemed to be the prime candidate for the infection route. Therefore, we examined the dietary history of the patients in the consultation sheet.

We found that, among 480 patients whose dietary habits were recorded, 326 patients (67.9 %) had a history or habit of ingesting raw or undercooked animal meat and/or liver. Consumed meat included chicken, beef, horse, wild boar and wild dear. Furthermore, when we took a close look at the seven patients younger than 10 years old, a 2-year-old girl had a history of ingesting slices of raw duck meat with her family, and two boys, aged 7 and 9 years old, each had a family history of ascarid LMS in their mother and grandparents, respectively. These facts strongly suggested that the major route of the infection was eating raw/undercooked paratenic host meat in Japan.

### Geographical distribution

**Fig. 6:**
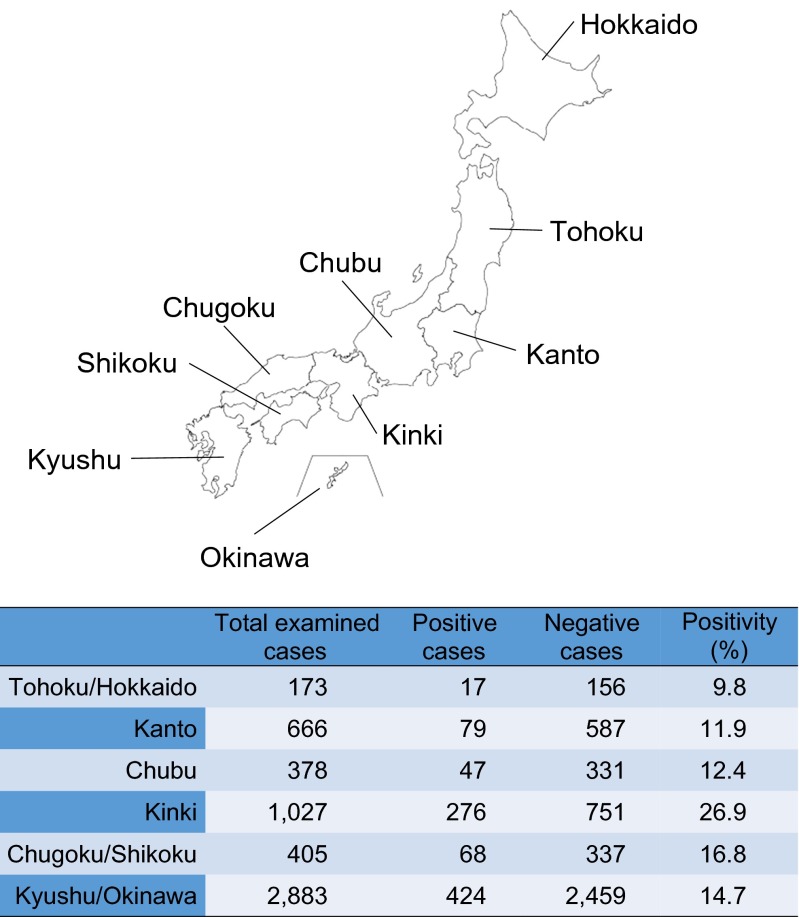
Positive sample ratio in different areas in Japan. The proportion of positive samples in the Kinki area was significantly higher than that in other areas

Another approach we took for a clue to the identification of the major infection route was geographical distribution. The numbers of patients from different areas of Japan were 424 from Kyushu Island (46.5 %), followed by 276 from Kinki and 79 from Kanto (Fig. 6). These data might, at first, give an impression that Kyushu was the most prevalent area for ascarid LMS. However, when the positive rate was calculated based on the number of total cases examined in each area, the highest rate was observed in Kinki (26.9 %), but not Kyushu. The positive rates of other areas were between 9.8 and 16.8 %, with an overall positive rate of 16.5 % (911/5532). Chi-square analysis demonstrated that the rate of positive patients in Kinki was significantly higher than that of other areas (*p* < 0.01).

## Discussion

Infection with *Toxocara* or pig *Ascaris* causes LMS, which is a typical parasitic zoonosis. Presentation may be acute, sub-acute or chronic, typically with respiratory manifestations associated with multiple nodular lesions in the lungs and/or liver. Occasionally, the central nervous system or the eyes are involved. Like in other parasitic worm infections with larval migration, peripheral blood eosinophilia is common and eosinophil infiltration may be seen in local fluids or tissues, which is a key to the correct diagnosis.

Eosinophilia is one of the famous keys for physicians to suspect worm infections [27, 28]. Although antibody testing is an indirect method for the identification of causative species, semi-quantitative microtitre plate ELISA with larva ES antigens combined with Western blotting can make a reliable discrimination between toxocariasis and ascariasis [29]. Based on the present results, approximately 90 % of ascarid LMS in Japan were probably caused by *Toxocara* nematodes (Fig. 3). As a Western blotting test detecting *A. suum*-specific antibody has recently been reported [30], a combination of *Toxocara* and *Ascaris* Western blotting would make diagnosis more reliable.

Regardless of the responsible species, however, it has been well-recognised that, in LMS, potent immune responses against migrating larvae result in the overproduction of white blood cells, especially eosinophils, and immunoglobulins [31]. In order to delineate the pathophysiology of ascarid LMS, we evaluated the intensity of immune responses in each disease type, by using peripheral blood eosinophil counts and serum total IgE.

We found that peripheral blood eosinophilia in the VLM, asymptomatic and miscellaneous types was prominent, often exceeding 3000/μl, suggesting that antigen load was relatively heavy in these groups. There were no significant differences in the number of eosinophils among these three types. On the other hand, the total IgE of the asymptomatic type was significantly higher than that of the miscellaneous type, with VLM in between (Table 1). These data suggest that the miscellaneous type is caused by an acute massive eosinophilia, which harmed various tissues such as the myocardium, intestinal mucosa or the skin, while the asymptomatic type is sub-acute or chronic. It could be possible that asymptomatic type cases were unnoticed when lung/liver eosinophilic infiltration was prominent.

OLM and NLM were characterised by weak eosinophilia and low serum IgE concentration. In OLM, no increase in eosinophilia or high IgE patients were demonstrated in residual analysis, which indicates that antigen load (number of infecting larvae) was too low to elicit immune responses. It is clear, as massive infection was not associated with eye infection, that migration into the eyes should not be a matter of probability. Instead, it should be associated with factors unique to younger people, because OLM was more common in younger patients (Fig. 4).

The present study based on 911 cases diagnosed during 2001 and 2015 revealed a unique character of this disease in Japanese patients. In short, this is primarily a disease of adult males. The majority of the patients were middle-aged males, and children patients were rare, which was the major difference from that reported from Europe, North American countries and tropical countries, where ascarid LMS was more common in children [12–18].

This difference in the epidemiology appears to be associated with food preference among Japanese people. They are definitely ‘sashimi lovers’ who have been enjoying sushi and sashimi of fish for a long time. People in Japan have a basic feeling that sashimi is the best way to enjoy natural delicacies [32]. Their eagerness can be seen in an anecdote that says a Japanese ichthyologist tried coelacanth sashimi captured off the coast of the Comoro Islands in the 1980s [33]. Amazingly or unfortunately, Japanese people have now extended their foodstuff territory for sashimi and sushi to animal meat, farmed as well as game meat. The fact that 67.9 % of the present patients had a history or habit of ingesting raw or undercooked animal meat and/or liver seems to suggest that they acquired infection by eating paratenic host meat.

Although the exact reason remains unclear, we found that the Kinki area in Japan had the highest positive rate for ascarid LMS (Fig. 6). We believe that this should also be associated with raw meat consumption. According to the family income and expenditure survey by the Japanese government, 8 out of the top 10 cities for average the consumption of beef per household based on expense were cities in the Kinki area [34]. Beef, especially expensive types, is often consumed raw in Japan. We also believe that the recent decrease of ascarid LMS patients diagnosed at our laboratory (Fig. 2) reflects reduced consumption of raw beef/bovine liver in the last few years. In 2011, a large food poisoning outbreak caused by enterohaemorrhagic *Escherichia coli* infection erupted in Japan, in which 181 patients were hospitalised with 21 acute encephalopathies and five deaths [35]. A law enforced in 2012 now prohibits restaurants from serving raw bovine liver to their customers.

In conclusion, LMS caused by *Toxocara* or *Ascaris* could be a disease of adult males, where raw or undercooked animal meat/liver are consumed, as in Japan. Healthcare specialists should draw public attention to the risk of raw or undercooked animal meat in Europe as well.
